# Characteristics of Children, Youth, and Young Adults With Diabetes: A Cross-Sectional Study in New Zealand Aotearoa

**DOI:** 10.1155/jdr/9968545

**Published:** 2024-12-10

**Authors:** Sara Mustafa, Ryan Paul, Rawiri Keenan, Dianna Magliano, Lynne Chepulis

**Affiliations:** ^1^Medical Research Centre, Te Huataki Waiora School of Health, University of Waikato, Hamilton, New Zealand; ^2^Te Whatu Ora Health New Zealand, Hamilton, New Zealand; ^3^School of Public Health and Preventive Medicine, Monash University, Melbourne, Australia

**Keywords:** adolescent, children, medication, New Zealand, retrospective study, Type 1 diabetes, Type 2 diabetes, youth

## Abstract

**Background:** The prevalence of diabetes mellitus among children, youth, and young adults is increasing, yet limited information is known about the characteristics and management of these groups with Type 1 (T1D) and Type 2 (T2D) diabetes in primary care. The aim of the study is to explore the characteristics of people with T1D and T2D aged < 25 years across the Auckland and Waikato regions of New Zealand.

**Methods**: Sociodemographic and clinical data were collected from electronic primary care records (February 2021–July 2022) of four primary healthcare organisations, with medication data sourced from the National Pharmaceutical dataset. Associations between sociodemographic and clinical data were conducted using chi-square and nonparametric ANOVA.

**Results:** Of 1198 patients, 72% had T1D and 28% had T2D. People with T1D were evenly distributed by gender but more commonly of European descent (66.7%) compared to other ethnic groups. A higher proportion of T2D was observed in females (58.2%) compared to males (41.6%) and among Māori (38.2% vs. 20.3% European; *p* < 0.001). Over 95% of individuals with T2D were overweight/obese. Overall, 9.5% and 23.9% of individuals with T1D and T2D, respectively, were at target for HbA1c, though median HbA1c was higher for Māori and Pasifika compared to other ethnicities (*p* < 0.001). In T1D, 94.7% of individuals were dispensed insulin and 7.5% and 4.4% were dispensed angiotensin-converting enzyme (ACE) inhibitors and statins, respectively. In T2D, medication use included metformin (84.9%), insulin (76.1%), and SGLT2i/GLP1RA (59.5%).

**Conclusions**: The increasing burden of diabetes among young individuals in New Zealand underscores the urgent need for comprehensive strategies to address obesity and socioeconomic disparities, especially among marginalised communities. Addressing socioeconomic factors such as affordable housing, living wages, and healthcare access may be important for improving diabetes outcomes, as these factors significantly influence overall childhood health and well-being.

## 1. Introduction

Diabetes mellitus is an increasing public health issue. It currently affects more than 300,000 people in Aotearoa New Zealand (NZ) and a disproportionate effect on Māori (the Indigenous people of NZ) [1]. Prevalence of Type 1 (T1D) and Type 2 (T2D) diabetes among children, young adults, and adults has been increasing globally [2–4]. In 2018, nearly 10% of all T2D cases in NZ were in children under 15 years old. [5] Prevalence of T2D have been reported to be higher in children and adolescents among minority populations globally [6–8], and have been reported to have increased five-fold among youth in some areas of NZ between 1995 and 2007 [9]. Māori children < 15 years are nearly twice as likely, and Pasifika children three times as likely, to develop T2D compared to the general population [10]. Additionally, Māori and Pasifika children are diagnosed at younger ages and with more severe complications [11].

Hyperglycaemia increases the likelihood of developing adverse long-term health outcomes [12]. Higher rates of peripheral neuropathy, diabetic kidney disease, and coronary heart disease have been reported to develop during adulthood among children with both T1D and/or T2D compared to those diagnosed in adulthood [13]. Importantly both T1D and T2D appear to be more aggressive in young adults compared to diabetes diagnosed in later life [14], and microvascular complications such as diabetic retinopathy have been reported in both children and youth with T2D [15]. Māori and other minority populations with either type of diabetes are at a greater risk of high glycaemia and are more likely to develop comorbidities than European [16, 17].

T1D is primarily managed via insulin to address the lack of endogenous insulin, whilst T2D is often managed via oral and injectable glucose-lowering therapies to address insulin resistance, with medication titration and insulin use as required. Since early 2021 in NZ, this may include the use of sodium-glucose cotransporter 2 inhibitors (SGLT2i) and glucagon-like peptide 1 receptor agonists (GLP1RA), although only recently (2023) have these been approved and/or clinically indicated for use in those at least 10 years old with T2D. [18]

Despite there being many studies describing T1D in children managed in secondary care in NZ [19], there is currently very little recent data describing diabetes in a cohort of children, youth, and young adults managed in primary care. However, given the increasing incidence and level of care required [1], there is a need to better understand the characteristics of this group, including the number of affected individuals, glycaemic control, and medication use. Thus, this study is aimed at exploring the characteristics of a cohort of patients with T1D and T2D aged < 25 years from across two large urban regions (Auckland and Waikato) in NZ.

## 2. Materials and Methods

### 2.1. Study Design and Participants

A cross-sectional study was conducted of clinical data collected for the 18-month period of February 2021–July 2022 from four primary healthcare organisations (PHOs), Pinnacle, ProCare, Hauraki, and National Hauora Coalition, across Waikato and Auckland regions in NZ. These PHOs all provided data (clinical and demographic) that had been extracted directly from their primary care datasets for all patients diagnosed with diabetes on February 1, 2021. This time period coincides with the funded availability of SGLT2i and GLP1RA, and this work was part of a larger study to explore the use of these new agents in T2D [20]. The PHOs were selected based on their involvement in other research projects, but collectively cover approximately 100% of the Waikato and 67% of the Auckland population.

All individuals enrolled in any of the four PHOs with a recorded diagnosis of either T1D or T2D (on Feb 1, 2021) and aged < 25 years were included in this study. Individuals were excluded if they had gestational or prediabetes or were 25 years and older. Diagnosis and type of diabetes were initially retrieved from the primary care records but were cross-referenced against hospital records from Te Whatu Ora Waikato and Starship Hospital Auckland, with prioritisation for diagnoses coded in secondary care.

### 2.2. Data Sources and Variables

Primary care participant data included demographic data and clinical information. Demographic data included participant age, ethnicity (Māori, Pasifika, European, Asian, and others), body mass index (BMI), and social deprivation data (NZDep18) [21]. Age was categorised according to NZ Ministry of Health national reports for children, youth, and young adults [22]. BMI was categorised based on the Centers for Disease Control and Prevention growth charts according to age and gender for participants < 20 years [23] and adult categories aged 20–24 years. Regional population data was accessed via the NZ Ministry of Health population webtool. Socioeconomic population data on youth < 25 years old in the Waikato and Auckland regions were included.

Clinical information included most recent results for blood pressure (BP), HbA1c, LDL-cholesterol (LDL-C), HDL-cholesterol (HDL-C), urinary albumin–creatinine ratio (UACR), and estimated glomerular filtration rate (eGFR). NHI-linked dispensed medication data was obtained from the National Pharmaceutical collection for the time period of February 2021–October 2022, allowing for the fact that prescriptions may be dispensed up to 90 days after being provided. Dispensed medication reviewed included metformin, vildagliptin, empagliflozin, dulaglutide, insulin, as well as statins, and angiotensin-converting enzyme (ACE) inhibitors. GLP1RA/SGLT2i was reported as a combined category given that NZ patients can only receive one or the other fully funded but may have switched between the two during the study period. Medication use in clinically indicated participants was defined for each medication: metformin (not clinically indicated for use for T1D; clinically indicated if HbA1c > 48 mmol/mol in individuals with T2D), ACEi (clinically indicated for use for T1D or T2D if UACR > 30 m/g or systolic BP > 130 mmHg and/or diastolic BP > 80 mmHg), statin (clinically indicated for use for T1D or T2D if LDL > 3.3 mmol/L), insulin (clinically indicated for use among for all with T1D and clinically indicated for use for T2D if HbA1c > 69 mmol/mol), vildagliptin (not clinically indicated for use for T1D or T2D) and sulfonylurea (not clinically indicated for use for T1D or T2D). Patients were deemed to be clinically indicated if they were above the clinical target for the required biomarker or if they were below the clinical target but were receiving the medication.

### 2.3. Statistical Analysis

Descriptive statistics were conducted to identify the proportion of affected individuals with T1D and T2D and to explore participant characteristics. The frequencies and percentages are presented for sociodemographic categorical variables, whilst the median and interquartile range were presented for continuous variables and clinical data. Associations between clinical data and sociodemographic characteristics were assessed using chi-square and Fisher's exact test where appropriate. Nonparametric ANOVA tests (Kruskal–Wallis) were conducted to assess differences in median across groups. Statistical significance was accepted at *p* < 0.05, and all analysis was conducted using IBM SPSS Version 29 (Armonk, NY).

### 2.4. Ethics

Ethics was obtained from the National Health and Disability Ethics Committee (ref 2022 AM8309).

## 3. Results

### 3.1. Demographics

A total of 1198 patients under the age of 25 were identified from a total diabetes cohort of 57,743 (0–75 years; main dataset compiled from the four PHOs) accounting for 2.1% of the diabetes population across these regions. This included 863 with T1D (72.0%) and 335 with T2D (28.0%; Table 1).

T1D was comparable between genders, and the median age for all patients with T1D was 16.0 years (interquartile range (IQR) = 12.0, 21.0) with more than two-thirds (66.7%) of patients identifying as NZ European ethnicity (vs. Māori (17.1%), Pasifika (8.1%), and Asian (6.0%); *p* < 0.001). Compared to the regional Auckland/Waikato region aged < 25 years, the burden of T2D was significantly higher among Māori (38.2% vs. 24.5%) and Pasifika (31.0% vs. 18.5%), but lower for Asian (9.3% vs. 24.0%) and European (20.3% vs. 33.0%). In contrast, the burden of T1D was higher among European youth (66.4% vs 33.0%) with lower rates observed for Māori, Pacific, and Asian youth (Table 1).

The number of patients diagnosed with T2D was higher in females than in males (58.4% vs. 41.6%; *p* = 0.003), increased with age (*p* < 0.001), and was more common in Māori (38.2%) and Pasifika (31.0%) compared to European (20.3%) and Asian (9.3%) (*p* < 0.001; Table 1).

### 3.2. Clinical Outcomes in T1D

In those with T1D, the overall median HbA1c was 70.0 mmol/mol (IQR = 63.0, 91.5; Table 1), but this was higher in those aged 20–25 compared to younger age groups (74.0 vs. 65.0 and 66.0 mmol/mol in 20–25 vs. 5–9 and 10–14, respectively; *p* = 0.012; Table S1). Higher HbA1c values were also observed among Pasifika (median = 85.0 mmol/mol, IQR = 74.3, 108) and Māori (median = 80.0 mmol/mol, IQR = 62.0, 100) compared to European and Asian (median = 67.0 and 62.0 mmol/mol, respectively; *p* < 0.001). Median HbA1c was higher in those with greater socioeconomic deprivation (81.0 vs. 63.0 mmol/mol in those living with the least deprivation; *p* = 0.006). Only 9.5% of patients with T1D were at target for HbA1c (48 mmol/mol) though more than 60% were at target for LDL-C and BP (Table 1).

The median systolic and diastolic BP among T1D was 123 mmHg (IQR = 110, 134) and 75.0 mmHg (IQR = 65.0, 83.5), respectively, whilst median HDL-C and LDL-C were 1.36 mmol/L (IQR = 1.11, 1.61) and 3.00 mmol/L (IQR = 2.30, 3.30), respectively. Median BP decreased whilst LDL-C values increased with advancing age (all *p* < 0.01; Table S1). There were no significant differences by patient characteristics for UACR and eGFR, though 8.6% of patients had microalbuminuria (UACR > 3 mg/mmol) and < 1% had macroalbuminuria (UACR > 30 mg/mmol).

### 3.3. Clinical Outcomes in T2D

The median HbA1c among individuals with T2D was 62.5 mmol/mol (IQR = 48.0, 88.0; Table 1), and this was higher in Pasifika (86.0 mmol/mol) and Māori (62.0 mmol/mol) compared to European (54.5 mmol/mol) and Asian (55.0 mmol/mol; *p* < 0.001; Table S2). No differences in HbA1c were observed by gender, age, BMI, or socioeconomic deprivation in T2D.

Hypertension was common in T2D. Males with T2D had significantly higher median systolic BP (median = 130 mmHg vs. 121 mmHg for females, *p* = 0.009; Table S2). Nearly a third of patients (35.8%) had microalbuminuria, and 35 (10.4%) had macroalbuminuria (Table 1) though this was significantly higher in males than in females (P <0.001). Nearly half of patients (44.6%) were at target for LDL-C, and neither median LDL-C or HDL-C levels differed by sociodemographic characteristics.

### 3.4. Medication Use

Overall, 94.7% of individuals with T1D were dispensed insulin during the study period. In addition, for those clinically indicated for medication, 7.5% were dispensed an ACEi/ARB (3.4% of all T1D), and 4.4% were dispensed a statin (0.8% of all T1D; Table 2). Overall, the use of metformin (5.6%) was more common in Pasifika (14.3%) and Māori (9.5%) with T1D compared to Asian (3.8%) and European (3.5%; *p* < 0.001), and among females (7.6%) compared to males with T1D (3.7%; *p* = 0.013). A small proportion of all patients with T1D were also dispensed vildagliptin (0.7%), sulfonylurea (0.2%), and GLP1RA/SGLT2i (0.1%).

The most common medication dispensed among individuals with T2D was metformin (68.7% of individuals; 84.9% clinically indicated), followed by GLP1RA/SGLT2i (32.8%), vildagliptin (29.9%) and insulin (24.8%). Overall metformin dispensing was highest in Pasifika (77.9%) and Asian (71.0%) groups compared to Māori (68.0%) and European (52.9%; *p* < 0.001; Table S3). Metformin use was also higher in individuals living in more deprived areas of NZ (78.6%–87.6%) compared to those living in the least deprived areas (56.7%–64.1%; *p* = 0.009). No difference in rates of dispensing of GLP1RA/SGLT2i, vildagliptin, and insulin was reported by sociodemographic characteristics. The median HbA1c of individuals with T2D who were dispensed GLP1RA/SGLT2i was higher than in those not receiving the medication (84.0 vs. 55.0 mmol/mol, *p* < 0.001).

A third of all clinically indicated patients with T2D were dispensed ACEi/ARB (36.5%) with dispensing rates increasing as age and sociodemographic deprivation increased (*p* < 0.001). ACEi/ARB dispensing was higher in Pasifika (34.6%), Māori (25.8%), and Asian (22.6%) compared to European (8.8%; *p* = 0.002). Sulfonylurea was dispensed to patients aged 15 years and over and was higher in Pasifika (16.3%) and Asian (12.9%) compared to European (4.4%) and Māori (3.1%; *p* < 0.001).

## 4. Discussion

This study analysed 1198 individuals younger than 25 years of age living in the Waikato/Auckland regions of NZ, with 68.4% having T1D and 26.6% having T2D. Although this represents only 2% of the total diabetes population aged < 75 years in these regions, it is an increasing and significant group of patients that require effective management and tailored healthcare strategies. T1D predominantly affected a higher proportion of Europeans compared to the underlying regional data, compared to Māori and Pasifika who have higher rates of T2D which aligns with the existing literature [24]. Whilst half of these patients had a healthy BMI, a high rate of overweight and obesity was also seen in those with T1D which is consistent with other studies [25].

T2D was also highly associated with obesity, as well as high socioeconomic deprivation in our study. These patients were also more likely to be Māori and Pasifika, which is consistent with the literature [26]. Interestingly, no differences in HbA1c levels were observed by gender, age, BMI, or socioeconomic deprivation in those with T2D. This suggests that whilst HbA1c control may not differ across these groups, the underlying risk factors such as obesity and socioeconomic deprivation are critical areas that need to be addressed. The high rates of overweight and obesity among those with T1D, even higher among those with T2D, highlight the failure of the NZ system to effectively address childhood obesity. Child poverty in NZ is a substantial concern, with approximately 12% of the child population residing in low-income housing, with notably higher proportions observed among Māori (14.5%) and Pasifika (19.5%) children [27]. Child poverty correlates with reduced access to food security and primary healthcare, elevating the risks of chronic conditions such as T2D and childhood mortality [26, 28]. The prevalence of childhood obesity has risen in NZ [29], a well-documented result of child poverty and associated to T2D development [30]. Low wages and high living costs may force many families to choose cheaper, calorie-dense foods over healthier options [31]. Addressing these issues requires a multifaceted approach, including stronger food regulations, better support for healthy lifestyles, comprehensive school programs, and economic policies that reduce poverty.

As with other literature, the majority of individuals with T1D and T2D did not meet the recommended HbA1c clinical target of < 48 mmol/mol [18]. In our study, Māori and Pasifika youth with T1D or T2D exhibited higher HbA1c levels compared to individuals of other ethnicities, aligning with the literature indicating a persistent equity gap in diabetes management in NZ [17, 26]. Research suggests that the causes for this are multifaceted. Key factors include lack of culturally safe care and financial barriers [26], reduced access to continuous glucose monitors [19], and psychological distress [32] in both Māori and Pasifika youth with T1D and T2D. Systemic issues related to colonisation and racism significantly contribute to these disparities. Historical colonisation has led to socioeconomic disadvantages, restricted access to quality healthcare, and institutional racism [33]. This leads to biases in healthcare and a lack of culturally safe care, which greatly affect how chronic conditions like diabetes are managed, leading to worse outcomes for Māori [34]. Further, young adults aged 20–25 years with T1D had a higher median HbA1c level than younger individuals, indicating a potential gap in the transition from paediatric to adult diabetes care. Diabetes management among young adults has been challenging [35], and the loss of free specialist-level care as an adult may contribute to the higher HbA1c levels.

The increasing proportion of insulin resistance among youth highlights the need to do more to address the determinants of early-onset T2D. Our study, consistent with the literature [36], reported better glycaemic control among youth with higher socioeconomic status. As individuals living in low socioeconomic areas are more likely to develop diabetes-related complications, this highlights the need for more targeted care and follow-up in these communities [37]. To mitigate the impact of socioeconomic deprivation on health outcomes, policy reforms that include investing in affordable housing, ensuring families earn a living wage sufficient to meet their needs, and enhancing access to comprehensive support services, such as healthcare and education, are necessary.

Clinically indicated use of metformin in youth with T2D was relatively high (84.9%). However, of concern, the use of other clinically indicated medications was relatively low, particularly in Māori and Pasifika patients. Such findings echo that seen in other studies [38] and support the need for urgent interventions to address prescribing inequities. This underprescription of necessary medications reflects broader issues of healthcare access and bias, where marginalised groups receive suboptimal care. Historical and systemic biases within the healthcare system have resulted in a lack of trust and engagement from these communities, further complicating diabetes management.

Interestingly, 30 patients with T2D had been dispensed SGLT2i/GLP1RA during our study. This is in line with clinical recommendations but is not reflective on current guidelines for use in youth between 12 and 16 years. Similarly, vildagliptin was dispensed for nine patients, and two patients received sulfonylureas, despite vildagliptin and other DPPIV inhibitors not being effective in those under 25 years and sulphonylureas possibly accelerating beta cell loss in this age group [18]. Management of diabetes is complex but is often more complicated in younger patients by the occurrence of more severe and/or progressive disease [15], the availability of medications for adult use yet different criteria for use in a younger cohort [39, 40]. Although these numbers are small, understanding the rationale behind these prescribing practices can reveal gaps in current treatment guidelines, highlight areas where existing protocols may not adequately address patient needs, and inform future updates to clinical guidelines to ensure they are based on the most recent evidence and best practices.

### 4.1. Limitations and Strengths

Whilst our study provides a comprehensive review of all patients under 25 with diabetes across four PHOs (and enrolled patient population of greater than one million), there are limitations. Firstly, our study is based predominantly on primary care records; a data source previously identified for its susceptibility to inaccuracies in diabetes coding, leading to misclassification bias [41]. This includes potential misclassification of type of diabetes as indicated by sulfonylurea dispensing in individuals with T1D, which is not clinically justified, as well as a lack of reputable data about the duration of diabetes, which is important when assessing and understanding diabetes management, particularly given the progressive nature of the disease that can be seen with early onset. Further, detailed specialist care records from secondary and tertiary care were not accessible due to the complexity of retrieving data across multiple regions and hospitals, exacerbated by the ongoing Health NZ restructuring. This limitation has led to an inability to assess the impact of specialist care access on patient outcomes, restricting the analysis to primary care data but should be explored in further studies.

Secondly, we note the importance of secondary specialist input for patients with T1D and T2D for all patients with T2D < 16 years. Whilst we were able to cross-reference NHIs with hospital lists to support coding of T1D and T2D, we were unable to retrieve clinical hospital records for these patients. However, we did use dispensing rather than prescribing data to support our interpretation of medication use, which does include prescribing from all sources across the country, thereby removing the primary care bias from this variable. Further, our data indicates that 95% of patients with T1D were dispensed insulin, suggesting potential errors in primary care coding despite cross-referencing with hospital datasets; however, young and newly diagnosed individuals may receive insulin in hospitals or diluted forms without formal prescriptions (and thus, do not have a dispensing record in our dataset), and a small subset may have other forms of diabetes warranting further study. Further, the Kruskal–Wallis test used may reduce sensitivity and power in detecting differences, such as HbA1c by deprivation quintile for T2D, leading to false negative bias.

Lastly, we note that whilst our numbers are representative of the Waikato/Auckland region and include a good representation of ethnicities, the proportion of diabetes reported in our study may not be indicative of the broader NZ context, as the study did not assess incidence or prevalence. However, the findings provide a valuable snapshot of diabetes and provide data to help optimise diabetes management in this cohort.

## 5. Conclusions

In conclusion, this study highlights the urgent need for comprehensive strategies to address diabetes among young individuals in the Waikato and Auckland regions. The disproportionate burden of diabetes among Māori and Pasifika communities, along with alarming rates of obesity and socioeconomic deprivation, underscores the imperative for targeted interventions prioritising equity and accessibility in healthcare. Moreover, the findings underscore systemic challenges in healthcare delivery, including disparities in medication access and the need for culturally competent care.

## Figures and Tables

**Table 1:** Sociodemographic characteristics and clinical measures by type of diabetes (all participants; *n* = 1198).

**Sociodemographic characteristics**	**Regional population** ^**a**^		**Type 1 diabetes**		**Type 2 diabetes**	
	**N**	**%**	**N**	**%**	**N**	**%**
Total	501,330	100	863	72.0	335	28.0
Gender						
Female	243,810	48.6	407	47.2	195	58.4
Male	257,520	51.4	456	52.8	139	41.6
*p* value			0.101		0.003	
Age in year median (IQR)			16.0 (12.0, 21.0)		21.0 (19.0, 23.0)	
< 10	195,600	39.0	120	13.9	2	0.6
10–14	104,760	20.9	210	24.3	22	6.6
15–19	98,140	19.6	244	28.6	82	24.5
20–24	102,830	20.5	286	33.1	229	68.4
*p* value			< 0.001		< 0.001	
Ethnicity						
Māori	120,020	24.5	148	17.1	128	38.2
Pasifika	92,890	18.5	70	8.1	104	31.0
Asian	122,850	24.0	52	6.0	31	9.3
European^b^	165,570	33.0	573	66.4	68	20.3
Others^b^	—	—	20	2.3	4	1.2
*p* value			< 0.001		< 0.001	
BMI^c^						
Underweight	—	—	4	2.7	2	2.1
Healthy	—	—	65	43.6	1	1.0
Overweight	—	—	33	22.8	12	12.4
Obese	—	—	46	30.9	82	84.5
*p* value			< 0.001		< 0.001	
Deprivation quintile						
1 (least deprived)	66,635	13.3	219	25.6	39	11.8
2	77,824	15.5	310	36.3	67	20.3
3	85,733	17.1	147	17.2	80	24.2
4	96,853	19.3	104	12.2	41	12.4
5 (most deprived)	174,286	34.8	74	8.7	103	31.2
Clinical measure median (interquartile range)						
HbA1c (mmol/mol)	—	—	70.0	(58.0, 85.0)	63.0	(49.0, 90.0)
LDL-C (mmol/L)	—	—	2.90	(2.23, 3.30)	2.70	(2.10, 3.50)
HDL-C (mmol/L)	—	—	1.38	(1.12, 1.62)	1.03	(0.87, 1.25)
Blood pressure (systolic/diastolic) (mmHg)	—	—	122.0/75.0	(110.0, 132.8)/(64.5, 83.0)	127.0/80.0	(120.0, 135.0)/(75.0, 86.0)
UACR (mg/mmol)	—	—	1.00	(0.50, 1.95)	2.80	(1.00, 17.2)
eGFR (mL/min/1.73 m^2^)	—	—	90.0	(90.0, 90.0)	90.0	(90.0, 90.0)
% patients at target						
HbA1c (≤ 48 mmol/mol)	—	—	57/600	9.5	58/242	23.9
LDL-C (≤ 2.6 mmol/L)	—	—	333/552	60.4	124/277	44.6
HDL-C (> 0.9 mmol/L)	—	—	27/563	4.8	80/294	27.2
Blood pressure (systolic BP ≤ 130 mmHg and diastolic BP ≤ 80 mmHg)	—	—	256/381	67.2	91/169	53.8
Renal profile (UACR < 3 mg/mmol and/or eGFR90mL/min)	—	—	517/561	92.3	212/274	77.1
Microalbuminuria (UACR > 3 mg/mmol)	—	—	74/860	8.6	120/336	35.8
Macroalbuminuria (UACR > 30 mg/mmol)	—	—	8/889	0.9	35/337	10.4

^a^Regional population data accessed via https://tewhatuora.shinyapps.io/populations-web-tool/.

^b^European and others are a combined category for regional population data.

^c^BMI was categorised on the CDC growth charts according to age and gender for < 20 years and adult categories for ages 20–24 years.

**Table 2:** Medication use among individuals < 25 years who are clinically indicated with T1D and T2D (*n* = 1198).

**Medication dispensed**	**Total**			**Type 1**			**Type 2**		
	**N** ** dispensed/clinically indicated**	**% of clinically indicated**	**% of total**	**N** ** dispensed/clinically indicated**	**% of clinically indicated**	**% of total**	**N** ** dispensed/clinically indicated**	**% of clinically indicated**	**% of total**
Metformin^a^	192/727	26.4	23.2	35	—	5.6	157/185	84.9	68.7
ACEi^b^	48/237	20.3	9.3	10/133	7.5	3.4	38/104	36.5	24.5
Statin^c^	13/169	7.7	3.0	4/90	4.4	0.8	9/79	11.4	8.7
Insulin^d^	900/972	92.6	75.1	817/863	94.7	94.7	83/109	76.1	24.8
SGLT2i/GLP1RA^a^	111/185	60.0	9.3	1	—	0.1	110/185	59.5	32.8
Vildagliptin^e^	106	—	8.8	6	—	0.7	100	—	29.9
Sulfonylurea^e^	31	—	2.6	2	—	0.2	29	—	8.7

^a^Not clinically indicated for use in T1D (used off-label); clinically indicated if HbA1c > 48 mmol/mol in T2D.

^b^Clinically indicated for use in T1D and T2D if UACR > 30 m/g or systolic BP > 130 mmHg and/or diastolic BP > 80 mmHg.

^c^Clinically indicated for use in T1D and T2D if LDL > 3.3 mmol/L.

^d^Clinically indicated for use among all T1D; clinically indicated for use in T2D if HbA1c > 69 mmol/mol.

^e^Not clinically indicated for use in T1D and T2D.

## Data Availability

The data that support the findings of this study are available on request from the corresponding author. The data are not publicly available due to privacy or ethical restrictions.

## Supporting Information

Supporting Information Additional supporting information can be found online in the Supporting Information section. Table S1: clinical outcomes by sociodemographic characteristics among individuals with T1D, including age, sex, ethnicity, socioeconomic status, and geographic location. Table S2: clinical outcomes by sociodemographic characteristics among individuals with T2D, covering similar variables as Table S1. Table S3: dispensing data among individuals with T1D and T2D by sociodemographic characteristics, detailing medication dispensing patterns.
