# Supplementary material for: Characteristics of Children, Youth, and Young Adults With Diabetes: A Cross-Sectional Study in New Zealand Aotearoa
Source: J Diabetes Res. 2024 Dec 10;2024:9968545. doi: 10.1155/jdr/9968545 (PMC11651759; doi:10.1155/jdr/9968545)
Supplement: Supporting Information — Additional supporting information can be found online in the Supporting Information section. Table S1: clinical outcomes by sociodemographic characteristics among individuals with T1D, including age, sex, ethnicity, socioeconomic status, and geographic location. Table S2: clinical outcomes by sociodemographic characteristics among individuals with T2D, covering similar variables as Table S1. Table S3: dispensing data among individuals with T1D and T2D by sociodemographic characteristics, detailing medication dispensing patterns. [file 9968545.f1.docx]

# **Supplementary Tables**

**Table S1. Clinical outcomes by sociodemographic characteristics among individuals with T1D.**

| Characteristics Median (IQR) | HbA1c (mmol/mol) | LDL (mmol/L) | HDL (mmol/L) | Blood pressure (systolic/diastolic) (mmHg) | UACR (mg/g) |
| --- | --- | --- | --- | --- | --- |
| Gender |  |  |  |  |  |
| Female | 69.0 (57.0, 84.0) | 2.55 (2.00, 3.20) | 1.52 (1.25, 1.77) | 115 (104, 123) / 70.0 (60.0, 74.0) | 1.00 (0.92, 1.58) |
| Male | 70.0 (58.0, 85.5) | 2.40 (1.90, 2.90) | 1.37 (1.18, 1.62) | 119 (110, 127) / 70.0 (61.5, 80) | 1.00 (0.63, 2.10) |
| *P value* | 0.911 |  | **<0.001** | 0.459 / 0.169 | 0.139 |
| Age in years |  |  |  |  |  |
| 1-4 | 66.5 (55.5, 75.3) | N/A | N/A | 135 (112, 141) / 80.0 (68.5, 84.5) | N/A |
| 5-9 | 65.0 (57.5, 77.3) | 2.20 (1.40, 2.58) | 1.43 (1.27, 1.71) | 132 (117, 142) / 80.0 (70.0, 89.0) | 1.05 (0.94, 2.05) |
| 10-14 | 66.0 (57.0, 80.0) | 2.20 (1.90, 2.80) | 1.50 (1.36, 1.78) | 120 (100, 130) /72.0 (65.0, 82.5) | 0.99 (0.99, 1.48) |
| 15-19 | 71.0 (57.5, 86.5) | 2.40 (2.00, 2.90) | 1.40 (1.19, 1.67) | 118 (108, 127) / 70.0 (65.0, 80.0) | 0.99 (0.50, 1.55) |
| 20-24 | 74.0 (58.0, 88.0) | 2.50 (2.00, 3.20) | 1.42 (1.18, 1.70) | 122 (110, 134) / 75.0 (68.0, 82.0) | 0.99 (0.99, 2.00) |
| *P value* | **0.012** | **0.025** | 0.058 | **0.004 / 0.019** | 0.619 |
| Ethnicity |  |  |  |  |  |
| Māori | 80.0 (62.0, 100) | 2.65 (2.03, 3.20) | 1.28 (1.06, 1.59) | 120 (110, 134) / 77.0 (70.0, 81.0) | 1.00 (0.99, 2.13) |
| Pasifika | 85.0 (74.3, 108) | 2.30 (2.00, 2.80) | 1.42 (1.14, 1.67) | 124 (112, 136) / 79.5 (62.5, 84,5) | 1.10 (0.99, 3.30) |
| Asian | 62.0 (53.5, 81.5) | 2.50 (1.90, 2.73) | 1.47 (1.28, 1.76) | 125 (105, 138) /75.0 (68.5, 87.0) | 0.99 (0.99, 1.38) |
| European | 67.0 (56.0, 80.0) | 2.40 (1.90, 3.00) | 1.47 (1.23, 1.71) | 120 (110, 132) / 71.0 (65.0, 80.0) | 0.99 (0.90, 1.60) |
| Other | 80.0 (61.0, 85.0) | 2.40 (1.83, 3.03) | 1.66 (1.44, 1.98) | 106 (100, 145) /65.0 (57.0, 95.0) | 0.99 (0.90, 1.00) |
| *P value* | **<0.001** | 0.405 | 0.052 | 0.757 / 0.251 | **0.020** |
| BMI |  |  |  |  |  |
| Underweight | 74.5 (55.0, 112) | 3.50 (2.53, 3.65) | 1.57 (1.30, 1.87) | 109 (88.0, 120)/62.0 (48.8, 76.0) | 2.00 (0.28, 7.03) |
| Healthy | 65.0 (54.0, 80.0) | 2.20 (1.70, 2.75) | 1.50 (1.27, 1.77) | 110 (102, 123) / 65.5 (60.0, 76.5) | 0.99 (0.99, 1.58) |
| Overweight | 74.5 (57.5, 85.8) | 2.60 (2.00, 2.95) | 1.34 (1.19, 1.62) | 118 (111, 124) / 72.5 (70.0, 80.0) | 0.99 (0.55, 2.20) |
| Obese | 75.0 (62.8, 90.3) | 2.65 (2.30, 3.10) | 1.30 (1.14, 1.54) | 120 (110, 125) / 70.0 (60.0, 80.0) | 1.00 (0.99, 2.00) |
| *P value* | 0.144 | 0.055 | 0.132 | 0.149 / 0.442 | 0.434 |
| Deprivation Quintile |  |  |  |  |  |
| 1 (least deprived) | 63.0 (56.0, 79.5) | 2.40 (2.00, 3.10) | 1.47 (1.25, 1.72) | 120 (107, 131) /71.0 (64.0, 80.0) | 0.99 (0.90, 1.53) |
| 2 | 70.0 (58.0, 84.0) | 2.50 (1.90, 3.08) | 1.42 (1.21, 1.70) | 120 (110, 134) / 75.0 (68.0, 82.0) | 0.99 (0.80, 1.70) |
| 3 | 69.0 (57.0, 90.0) | 2.30 (1.80, 2.90) | 1.43 (1.13, 1.69) | 120 (110, 135) / 72.0 (70.0, 84.0) | 1.00 (0.99, 2.30) |
| 4 | 72.5 (62.3, 84.8) | 2.40 (1.90, 2.70) | 1.43 (1.18, 1.67) | 116 (102, 129) / 70.0 (60.0, 78.3) | 0.99 (0.99, 1.98) |
| 5 (most deprived) | 81.0 (65.0, 98.0) | 2.60 (1.95, 3.05) | 1.40 (1.10, 1.61) | 123 (110, 129) / 78.5 (68.5, 80.0) | 0.99 (0.99, 1.55) |
| *P value* | **0.006** | 0.380 | 0.500 | 0.841 / **0.046** | 0.947 |

**Table S2. Clinical outcomes by sociodemographic characteristics among individuals with T2D.**

| Characteristics  Median (IQR) | HbA1c (mmol/mol) | LDL (mmol/L) | HDL (mmol/L) | Blood pressure (systolic/diastolic) (mmHg) | UACR (mg/g) |
| --- | --- | --- | --- | --- | --- |
| Gender |  |  |  |  |  |
| Female | 63.5 (49.3, 86.0) | 2.70 (2.10, 3.50) | 1.10 (0.92, 1.31) | 121 (112, 130) / 80.0 (70.0, 85.0) | 2.10 (0.99, 9.10) |
| Male | 64.5 (48.0, 98.3) | 2.70 (2.20, 3.50) | 1.03 (0.88, 1.15) | 130 (120, 140) / 80.0 (70.0, 88.0) | 4.50 (1.40,19.2) |
| *P value* | 0.896 | 0.922 | 0.126 | **0.009 /** 0.995 | **0.021** |
| Age in years |  |  |  |  |  |
| 10-14 | 67.0 (44.0, 89.5) | 2.40 (2.15, 3.35) | 1.08 (0.89, 1.21) | 126 (106, 136) / 79.0 (69.3, 83.8) | 2.70 (1.00, 5.90) |
| 15-19 | 57.0 (44.3, 86.0) | 2.50 (2.20, 3.40) | 1.09 (0.92, 1.31) | 120 (110, 131) / 80.0 (70.0, 87.0) | 2.50 (1.00, 7.95) |
| 20-24 | 66.5 (51.3, 95.0) | 2.80 (2.10, 3.50) | 1.06 (0.90, 1.20) | 125 (118, 137) / 80.0 (70.5, 85.5) | 2.70 (1.00, 14.5) |
| *P value* | 0.264 | 0.168 | 0.538 | 0.627 / 0.418 | 0.446 |
| Ethnicity |  |  |  |  |  |
| Māori | 62.0 (47.0, 88.5) | 2.70 (2.10, 3.50) | 1.04 (0.89, 1.20) | 124 (113, 134) / 80.0 (70.0, 86.0) | 3.60 (1.20, 13.6) |
| Pasifika | 86.0 (61.0, 102) | 2.70 (2.20, 3.30) | 1.09 (0.92, 1.27) | 126 (117, 130) / 80.0 (70.8, 90.0) | 5.40 (1.60, 18.6) |
| Asian | 55.0 (46.0, 73.0) | 2.80 (2.35, 3.60) | 1.07 (0.97, 1.35) | 126 (120, 134) / 80.0 (70.0, 83.0) | 1.00 (0.99, 2.90) |
| European | 54.5 (45.0, 67.3) | 2.90 (2.10, 3.60) | 1.08 (0.93, 1.35) | 125 (117, 137) / 80.0 (70.0, 86.0) | 1.40 (0.99, 3.80) |
| Other | 55.0 (42.5, 64.0) | 2.55 (1.68, 3.35) | 1.10 (0.97, 1.22) | 120 (115, 120) / 70.0 (70.0, 70.0) | 3.10 (1.60, 4.60) |
| *P value* | **<0.001** | 0.453 | 0.910 | 0.561 / 0.685 | **0.006** |
| BMI |  |  |  |  |  |
| Overweight | 83.0 (57.0, 94.0) | 3.10 (2.30, 3.60) | 1.16 (0.97, 1.52) | 129 (115, 132) | 2.00 (0.90, 3.20) |
| Obese | 70.5 (52.0, 96.5) | 2.90 (2.30, 3.50) | 1.04 (0.90, 1.22) | 120 (120, 130) | 4.40 (1.20, 17.8) |
| *P value* | 0.370 | 0.539 | 0.192 | 0.619 / 0.642 | 0.058 |
| Deprivation Quintile |  |  |  |  |  |
| 1 | 59.0 (42.8, 73.8) | 2.80 (2.30, 3.40) | 1.10 (0.97, 1.40) | 126 (117, 139) / 80.0 (70.0, 86.5) | 1.15 (0.99, 2.45) |
| 2 | 55.5 (44.8, 77.5) | 2.65 (2.10, 3.60) | 1.05 (0.83, 1.20) | 125 (110, 134) / 80.0 (70.0, 86.0) | 2.60 (0.99, 13.2) |
| 3 | 68.0 (49.8, 88.3) | 3.00 (2.30, 3.60) | 1.10 (0.92, 1.30) | 123 (119, 138) / 80.0 (71.0, 85.5) | 3.60 (1.40, 12.2) |
| 4 | 74.0 (52.0, 101) | 2.60 (2.00, 3.20) | 1.09 (0.92, 1.16) | 126 (113, 130) / 80.0 (72.5, 84.5) | 3.25 (0.99, 12.8) |
| 5 | 70.0 (52.0, 100) | 2.70 (2.10, 3.30) | 1.05 (0.89, 1.16) | 120 (113, 130) / 78.0 (70.0, 86.0) | 4.20 (1.45, 16.6) |
| *P value* | 0.162 | 0.288 | 0.530 | 0.587 / 0.836 | **0.026** |

**Table S3. Dispensing data among individuals with T1D and T2D by sociodemographic characteristics**

| Demographic characteristics | Metformin | | Insulin | | | GLP1RA/  SGLT2i | | Vildagliptin | | Sulfonylurea | | Statin | | ACEi | |
| --- | --- | --- | --- | --- | --- | --- | --- | --- | --- | --- | --- | --- | --- | --- | --- |
|  | **N** | **%** | **N** | | **%** | **N** | **%** | **N** | **%** | **N** | **%** | **N** | **%** | **N** | **%** |
| Type 1 Diabetes |  |  |  | |  |  |  |  |  |  |  |  |  |  |  |
| Total | **48** | **5.6** | **817** | | **94.7** | **1** | **0.1** | **6** | **0.7** | **2** | **0.2** | **7** | **0.8** | **29** | **3.4** |
| Age | | | | | | | | | | | | | | | |
| 1-4 | 0 | 0.0 | 20 | | 76.9 | 0 | 0.0 | 0 | 0.0 | 0 | 0.0 | 0 | 0.0 | 0 | 0.0 |
| 5-9 | 1 | 1.1 | 80 | | 85.1 | 0 | 0.0 | 0 | 0.0 | 0 | 0.0 | 0 | 0.0 | 0 | 0.0 |
| 10-14 | 9 | 4.3 | 204 | | 97.1 | 0 | 0.0 | 0 | 0.0 | 1 | 0.5 | 0 | 0.0 | 2 | 1.0 |
| 15-19 | 17 | 6.9 | 234 | | 94.7 | 1 | 0.4 | 3 | 1.2 | 1 | 0.4 | 1 | 0.4 | 4 | 1.6 |
| 20-24 | 21 | 7.3 | 279 | | 97.6 | 0 | 0.0 | 3 | 1.0 | 0 | 0.0 | 6 | 2.1 | 23 | 8.0 |
| *P value* | 0.077 | | **<0.001** | | | 0.669 | | 0.522 | | 0.649 | | 0.112 | | **<0.001** | |
| Gender | | | | | | | | | | | | | | | |
| Female | 31 | 7.6 | 389 | | 95.6 | 1 | 0.2 | 4 | 1.0 | 1 | 0.2 | 3 | 0.7 | 11 | 2.7 |
| Male | 17 | 3.7 | 428 | | 93.9 | 0 | 0.0 | 2 | 0.4 | 1 | 0.2 | 4 | 0.9 |  | 3.9 |
| *P value* | **0.013** | | 0.262 | | | 0.472 | | 0.429 | | -- | | 0.819 | | 0.311 | |
| Ethnicity | | | | | | | | | | | | | | | |
| Māori | 14 | 9.5 | 138 | | 93.2 | 1 | 0.7 | 1 | 0.7 | 1 | 0.7 | 3 | 2.0 | 5 | 3.4 |
| Pasifika | 10 | 14.3 | 67 | | 95.7 | 0 | 0.0 | 2 | 2.9 | 0 | 0.0 | 3 | 4.3 | 5 | 7.1 |
| Asian | 2 | 3.8 | 47 | | 90.4 | 0 | 0.0 | 0 | 0.0 | 0 | 0.0 | 0 | 0.0 | 1 | 1.9 |
| European | 20 | 3.5 | 545 | | 95.1 | 0 | 0.0 | 3 | 0.5 | 1 | 0.2 | 1 | 0.2 | 18 | 3.1 |
| Other | 2 | 10.0 | 20 | | 100.0 | 0 | 0.0 | 0 | 0.0 | 0 | 0.0 | 0 | 0.0 | 0 | 0.0 |
| *P value* | **<0.001** | | 0.407 | | | 0.336 | | 0.260 | | 0.559 | | **0.007** | | 0.478 | |
| Deprivation Quintile | | | | | | | | | | | | | | | |
| 1 | 10 | 4.6 | 212 | | 96.8 | 0 | 0.0 | 1 | 0.5 | 0 | 0.0 | 0 | 0.0 | 8 | 3.7 |
| 2 | 12 | 3.9 | 292 | | 94.2 | 0 | 0.0 | 1 | 0.3 | 1 | 0.7 | 1 | 0.3 | 8 | 2.6 |
| 3 | 10 | 6.8 | 136 | | 92.5 | 1 | 0.7 | 1 | 0.7 | 1 | 1.0 | 4 | 2.7 | 7 | 4.8 |
| 4 | 6 | 5.8 | 100 | | 96.2 | 0 | 0.0 | 1 | 1.0 | 0 | 0.0 | 0 | 0.0 | 5 | 4.8 |
| 5 | 9 | 12.2 | 69 | | 93.2 | 0 | 0.0 | 2 | 2.7 | 33 | 30.6 | 1 | 1.4 | 1 | 1.4 |
| *P value* | 0.067 | | 0.372 | | | 0.381 | | 0.197 | | 0.145 | | **0.019** | | 0.538 | |
| Type 2 Diabetes | | | | | | | | | | | | | | | |
| Total | **230** | **68.7** | | **83** | **24.8** | **110** | **32.8** | **100** | **29.9** | **29** | **8.7** | **29** | **8.7** | **82** | **24.5** |
| Age | | | | | | | | | | | | | | | |
| 5-9 | 2 | 100.0 | | 0 | 0.0 | 0 | 0.0 | 1 | 50.0 | 0 | 0.0 | 1 | 50.0 | 1 | 50.0 |
| 10-14 | 15 | 68.2 | | 9 | 40.9 | 8 | 36.4 | 4 | 18.2 | 0 | 0.0 | 0 | 0.0 | 3 | 13.6 |
| 15-19 | 52 | 63.4 | | 20 | 24.4 | 25 | 30.5 | 20 | 24.4 | 6 | 7.3 | 1 | 1.2 | 10 | 12.2 |
| 20-24 | 161 | 70.3 | | 54 | 23.6 | 77 | 33.6 | 75 | 32.8 | 23 | 10.0 | 27 | 11.8 | 68 | 29.7 |
| *P value* | 0.561 | | | 0.293 | | 0.822 | | 0.232 | | 0.435 | | **<0.001** | | **<0.001** | |
| Gender | | | | | | | | | | | | | | | |
| Female | 139 | 71.3 | | 55 | 28.2 | 67 | 34.4 | 55 | 28.2 | 18 | 9.2 | 11 | 5.6 | 37 | 19.0 |
| Male | 90 | 64.7 | | 28 | 20.1 | 43 | 30.9 | 45 | 32.4 | 11 | 7.9 | 18 | 12.9 | 45 | 32.4 |
| *P value* | 0.205 | | | 0.093 | | 0.512 | | 0.412 | | 0.673 | | **0.019** | | **0.005** | |
| Ethnicity | | | | | | | | | | | | | | | |
| Māori | 87 | 68.0 | | 33 | 25.8 | 37 | 28.9 | 34 | 26.6 | 4 | 3.1 | 14 | 10.9 | 33 | 25.8 |
| Pasifika | 81 | 77.9 | | 30 | 28.8 | 43 | 41.3 | 38 | 36.5 | 17 | 16.3 | 11 | 10.6 | 36 | 34.6 |
| Asian | 22 | 71.0 | | 3 | 9.7 | 9 | 29.0 | 9 | 29.0 | 4 | 12.9 | 1 | 3.2 | 7 | 22.6 |
| European | 36 | 52.9 | | 17 | 25.0 | 20 | 29.4 | 18 | 26.5 | 3 | 4.4 | 39 | 4.4 | 6 | 8.8 |
| Other | 4 | 100.0 | | 0 | 0.0 | 1 | 25.0 | 1 | 25.0 | 1 | 25.0 | 0 | 0.0 | 0 | 0.0 |
| *P value* | **0.009** | | | 0.201 | | 0.290 | | 0.506 | | **<0.001** | | 0.421 | | **0.002** | |
| Deprivation Quintile | | | | | | | | | | | | | | | |
| 1 | 25 | 64.1 | | 9 | 23.1 | 12 | 30.8 | 8 | 20.5 | 1 | 2.6 | 1 | 2.6 | 6 | 15.4 |
| 2 | 38 | 56.7 | | 15 | 22.4 | 16 | 23.9 | 15 | 22.4 | 3 | 4.5 | 7 | 10.4 | 10 | 14.9 |
| 3 | 46 | 57.5 | | 21 | 26.3 | 29 | 36.3 | 24 | 30.0 | 5 | 6.3 | 7 | 8.8 | 12 | 15.0 |
| 4 | 36 | 87.8 | | 11 | 26.8 | 13 | 31.7 | 14 | 34.1 | 6 | 14.6 | 3 | 7.3 | 13 | 31.7 |
| 5 | 81 | 78.6 | | 26 | 25.2 | 36 | 35.0 | 38 | 36.9 | 13 | 12.6 | 10 | 9.7 | 39 | 37.9 |
| *P value* | **<0.001** | | | 0.977 | | 0.537 | | 0.186 | | 0.105 | | 0.692 | | **<0.001** | |

Abbreviations: GLP1RA/SGLT2i, Glucagon-like peptide 1 receptor agonists/sodium-glucose cotransporter 2 inhibitors; ACEi, angiotensin-converting enzyme inhibitors; BMI, body mass index
